# *De *N*ovo*-Designed Miniprotein Inhibits the Enzymatic Activity of the SARS-CoV‑2 Main Protease

**DOI:** 10.1021/acs.jcim.5c01708

**Published:** 2025-10-08

**Authors:** Tayná E. Lima, Emerson G. Moreira, Danilo F. Coêlho, Carlos H. B. Cruz, Rafael Dhalia, Bruno H. S. Leite, Lícya S. Xavier, Marta Perez Illana, Gabriel L. Wallau, Isabelle F. T. Viana, Roberto D. Lins

**Affiliations:** † Department of Virology, Aggeu Magalhães Institute, Oswaldo Cruz Foundation, Recife 50740-465, Brazil; ‡ Department of Fundamental Chemistry, 28116 Federal University of Pernambuco, Recife 50740-540, Brazil; § 4919 Institute of Structural and Molecular Biology, University College London, London WC1E 7HX, U.K.; ∥ Department of Entomology, Aggeu Magalhães Institute, Oswaldo Cruz Foundation, Recife 50740-465, Brazil; ⊥ Genomic Network Fiocruz, Oswaldo Cruz Foundation, Recife 50740-465, Brazil; # Graduate Program in Materials Science, Federal University of Pernambuco, Recife 50740-540, Brazil

## Abstract

Targeting viral proteases is a well-established antiviral strategy and a promising approach that has been actively explored against SARS-CoV-2. The SARS-CoV-2 main protease (M^pro^) is essential for viral replication and functions as a homodimer, making its dimerization interface an attractive therapeutic target. In this study, we report the rational design of HB3-Core25, a miniprotein computationally engineered to disrupt M^pro^ dimerization and inhibit its catalytic activity. *In vitro* production followed by biophysical characterization showed that HB3-Core25 folds into a compact trimeric helical bundle, exhibiting high solubility and thermal stability. Biophysical assays confirmed binding to M^pro^ with a dissociation constant (*K*_D_) of 0.567 μM and the lowest IC50 reported to date for the dimer interface. Functional assays further demonstrated inhibition of M^pro^ catalytic activity, with 51.1%. These findings highlight HB3-Core25 as a stable inhibitor of M^pro^ activity by interfering with its dimerization, offering a complementary strategy to classical active-site inhibition in antiviral drug development.

## Graphical abstract

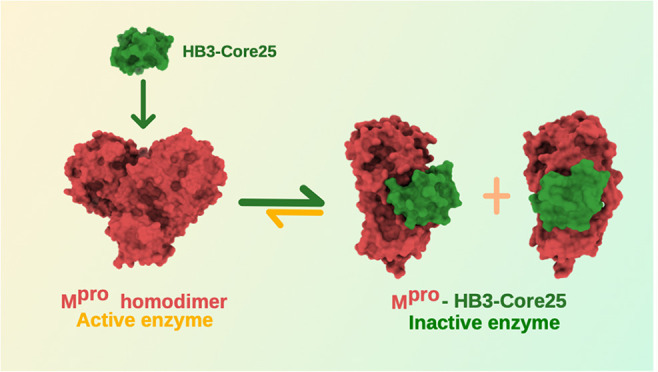

## Introduction

Viruses often rely on proteases to cleave large polyproteins into functional units, enabling the production of mature viral proteins essential for replication and assembly of new infectious particles. This mechanism not only ensures efficient replication but also allows viruses to maintain compact genomes.^1,2^ Owing to their indispensable role in the viral life cycle, viral proteases have emerged as well-established therapeutic targets, with both competitive and allosteric inhibitors successfully deployed against pathogens such as the human immunodeficiency virus (HIV) and hepatitis C virus (HCV). In the wake of the COVID-19 pandemic, similar antiviral strategies have been redirected toward combating Severe Acute Respiratory Syndrome Coronavirus 2 (SARS-CoV-2), the causative agent of the disease.^3,4^

SARS-CoV-2 encodes two polyproteins, pp1a and pp1ab, which are cleaved into 16 nonstructural proteins (NSPs) by two viral proteases: the papain-like protease (PL^pro^) and the main chymotrypsin-like protease (M^pro^). PL^pro^ cleaves the junctions between NSP1 and NSP4, whereas M^pro^ processes 11 additional cleavage sites, spanning NSP4–NSP16, including autocleavage at its own N- and C-terminal ends.^5^ Because of its crucial role in viral replication, M^pro^ has become a primary target in the development of antiviral drugs.^6,7^ It is a cysteine protease that operates as a homodimer with catalytic activity driven by a dyad composed of histidine 41 and cysteine 145.^8,9^ While significant efforts have been applied toward the discovery of effective antivirals for SARS-CoV-2, the vast majority of molecules that have completed clinical trials were originally developed for other infectious and inflammatory diseases and were repurposed for COVID-19.^10^ Various approaches have been explored, including chemically modified and structure-guided designed peptides targeting the catalytic site or the dimerization interface of M^pro^, although drug development has primarily focused on competitive inhibitors.^4,7,10–12^

Meanwhile, the N-terminal region of M^pro^, commonly referred to as the N finger, is particularly important for maintaining its homodimeric structure, contributing approximately 39% of the interaction interface.^4,8^ Prior studies have attempted to interfere with this interaction using peptides that mimic the N-terminal sequence, e.g., SGFRKMAF (N8 peptide) for SARS-CoV-1 and SGFRKMAFPS for SARS-CoV-2. These peptides successfully reduced enzymatic activity by up to 46.2%; however, they required high concentrations (IC_50_ ≥ 500 μM), likely due to their conformational flexibility.^13,14^ Although these peptides demonstrate a proof-of-concept for disrupting M^pro^ dimerization, their limited potency and poor stability diminish their therapeutic potential.

To address these challenges, computational protein design offers a compelling alternative for targeting M^pro^’s dimerization interface.^15–17^ Small molecules and peptides have played central roles in antiviral drug development due to their oral bioavailability and ease of synthesis. However, they often struggle to target flat protein–protein interfaces, such as that of M^pro^ dimerization. Peptides can engage larger surfaces but are limited by low stability and rapid degradation. In contrast, synthetic proteins, particularly miniproteins, offer improved stability, resistance to proteolysis, and customizable pharmacokinetics. Miniproteins are compact, *de novo*-designed scaffolds (<50 residues) displaying properties enabling high-affinity and selective targeting of challenging surfaces, including the well-defined secondary structure, cooperative folding, and sequestered hydrophobic cores.^18^

In this study, we employed *de novo* design to generate a miniprotein that selectively binds to this critical region of SARS-CoV-2 M^pro^. Through structural and thermal stability characterization, along with *in vitro* functional assays, we demonstrate that this miniprotein exhibits a high binding affinity and effectively inhibits M^pro^ catalytic activity. Our findings lay the ground for a new class of antiviral agents that may complement existing therapies in the ongoing fight against COVID-19.

## Experimental Section

### Computational Procedures

The computational workflow comprised four key steps: (1) evaluation of N8 peptide-M^pro^ interactions; (2) *de novo* design of miniprotein inhibitors; (3) stability assessment of designed inhibitors; and (4) analysis of inhibitor stability and interface dynamics.

### Structure Preparation

The monomeric structure of SARS-CoV-2 M^pro^ and its N8-derived peptide complex (M^pro^-N8) were modeled based on the crystallographic structure of the M^pro^ homodimer (PDB ID: 7ALI, resolution: 1.65 Å). Crystallographic water molecules, ligands, and ions were removed prior to energy minimization, which was performed using the FastRelax protocol in the Rosetta suite (v3.13 ^19^) with the scoring function described elsewhere.^20^

### Alanine Scanning

Binding hotspots within the N8 peptide were identified using a consensus approach across three alanine scanning methodologies: (1) FoldX v5;^21^ (2) MutaBind2 web server;^2^ and (3) Rosetta’s Flex ddG protocol.^3^ The contribution of each residue to binding energy (ΔΔG_bind_) was calculated using the equation ΔΔ*G*_bind_ = Δ*G*_ala_ – Δ*G*_wt_, where Δ*G*_wt_ and Δ*G*_ala_ refer to the binding free energies of the wild-type and alanine-mutated peptides, respectively. Hotspot criteria were defined according to the criteria of each method used: (1) Foldx: +0.92 kcal/mol < ΔΔ*G*_bind_ ≤ +1.84 kcal/mol;^22,23^ (2) MutaBind2: ΔΔ*G*_bind_ ≥ +1.5 kcal/mol;^24^ and (3) Flex ddG: ΔΔ*G*_bind_ ≥ +1.7 kcal/mol.^25^ A residue was classified as a binding hotspot if it met the threshold in at least two of the three methods.

### De Novo Design of Miniprotein

The *de novo* design strategy, adapted from previous studies,^26,27^ involved four stages: (1) motif binding design; (2) backbone design; (3) sequence design (monomer and interface); and (4) sequence-structure compatibility prediction. A 16-residue α-helix scaffold (H16) composed of valines (V) was generated using RosettaRemodel^28^ as a scaffold due to its structural compatibility with the binding groove of M^pro^. Structural alignment of H16 Cα atoms with those of N8 hotspot residues guided motif positioning (Figure 1A). Based on the alanine scanning results from N8 in complex with M^pro^ (Figure S1), valine residues in H16 at the corresponding positions of R4 and M6 were mutated to R and M, respectively, using Pymol’s mutagenesis tool. Optimal side chain rotamers for these residues were manually selected prior to starting the sequence design protocol. These residues were not mutated during the design.

**Figure 1:**
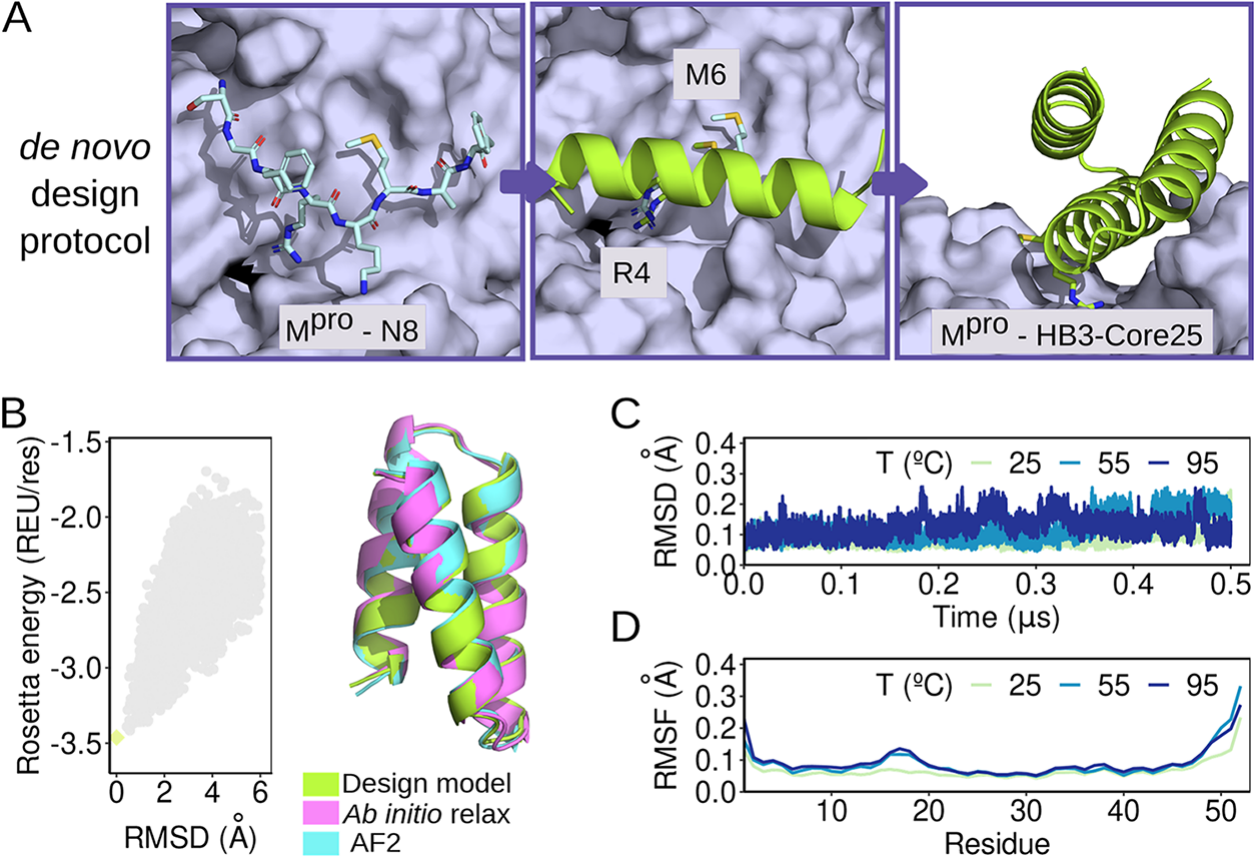
Computational *de novo* design of the M^pro^-binding miniprotein. (A) Design protocol of the M^pro^-binding protein backbone. M^pro^ surface is shown in light purple, while the N8 peptide is depicted in pink licorice mode. The H16 residue and the transplanted R and M residues are shown in green. The backbone model of the miniprotein mimetic derived from the N8 peptide is also shown in green cartoon mode. (B) Assessment of the prediction quality. Results of the *ab initio* relaxation simulations are shown in the plot (left). The horizontal axis represents the Rosetta energy per residue, while the vertical axis represents the root-mean-square deviation (RMSD) of the structures relative to the design model. Gray dots correspond to all generated structures; the light green dot indicates the lowest-energy structure. On the right side, low-scoring models from the folding simulations with *ab initio* relax (pink) are superimposed on the models predicted by AF2 (cyan) and the design model (light green) in cartoon mode. (C) RMSD and (D) root-mean-square fluctuation (RMSF) obtained by classical molecular dynamics simulations, having the designed coordinates as the reference structure, in three temperatures: 25 °C (green), 55 °C (blue), and 95 °C (dark blue).

Backbone design was carried out using Rosetta’s BluePrintBDR protocol via sequence-independent fragment assembly simulations.^29^ A three-helix bundle topology with 2–3 residue loops was selected, based on previous studies.^30^ Backbone torsion angles were defined using the ABEGO notation. For each blueprint file, 15 000 models were generated using the *fldcen.wts* weight set. Designs were selected based on (1) ≥20% of residues forming a hydrophobic core and (2) absence of steric clashes with M^pro^.

The sequence design was performed in two steps: full monomer sequence design followed by interface optimization (residues within 10 Å of M^pro^ were refined for enhanced binding affinity). The FastDesign protocol generated 50 000 sequences. Additional scripts (RelaxScripts, MonomerDesign2019, and InterfaceDesign2019) were used to ensure incorporation of bulky residues at the hydrophobic core and interface.^31^ Rosetta's LayerDesign TaskOperation was applied to restrict the sequence space. Designs were filtered based on monomer and interface criteria. Monomer metrics included: energy per residue ≤ −3 kcal/mol, shape complementarity (SS-Sc) ≥ 0.8, buried nonpolar area residue per (nspa) ≥ 50 Å^2^, ≤5 alanines overall, ≤3 alanines in the hydrophobic core, and ≥20% hydrophobic core residues. Interface metrics included: ddG ≤ −40 kcal/mol, buried solvent-accessible surface area (SASA) ≤ 1200 Å^2^, interface energy density (dG separated × 100/dSASA), buried unsatisfied hydrogen bonds (ΔInsatLH) ≤ 10, shape complementarity (Sc) ≥ 0.6, molecular contact area ≥ 450, and PackStat ≥ 0.65.^15^ Structure prediction was performed to validate sequence-structure compatibility using both *ab initio* relaxation (Rosetta v3.13) and AlphaFold2 (AF2) via ColabFold.^32^ In the Rosetta protocol, 3- and 9-residue fragments generated with Robetta^33^ were used to build the protein structure for simulations (25 000–30 000 total). The generated structures were compared to the original design model. Folding success was defined by the emergence of a funnel in the energy vs RMSD plot. All Rosetta Scripts from backbone generations and sequence design can be found in the Supporting Information.

### Molecular Dynamics (MD) Simulations

MD simulations were performed with GROMACS 2019.4 ^34^ using the CHARMM36m force field^35^ to assess the structural stability of the HB3-Core25 miniprotein, its complex with M^pro^, and the M^pro^ dimer. Protonation states were assigned with H++ server^36^ at pH 7.4 and 0.15 M ionic strength. Systems were solvated with TIP3P water in a cubic box and neutralized with counterions (achieving a final ionic concentration of 150 mM). Energy minimization was performed using 5000 steps of the Steepest Descent algorithm (maximum atomic force ≤ 100 kJ mol^–1^ nm^–2^). Systems were heated (NVT ensemble, 1 ns) and equilibrated in five 1 ns NPT steps, reducing positional restraints from 5000 kJ·mol^–1^ to 1000 kJ mol^–1^ nm^–2^. Production runs were performed by using the NPT ensemble: 0.5 μs for the monomer and 1.0 μs for complexes. Periodic boundary conditions were applied in all directions (*x*, *y*, and *z*) during all simulation stages. The *V-*rescale maintained a temperature constant with a coupling constant of 0.4 ps.^37^ Pressure was set to 1 bar using the Parrinello–Rahman barostat^38^ with a relaxation time of 2.0 ps and a compressibility constant of 4.6 × 10^–5^ bar^–1^. Long-range electrostatics were handled using the PME method, while van der Waals interactions and short-range electrostatics were truncated at 1.2 nm cutoff radii. The LINCS algorithm was used to constrain bonds involving hydrogen atoms.^39,40^ The leapfrog algorithm,^41^ with a 2.0 fs integration time step, was used for numerical integration equations of motion. Simulation box sizes were 5.50 nm^3^ for the M^pro^ monomer; 9.98 nm^3^ for the M^pro^ homodimer, and 9.80 nm^3^ for the M^pro^-HB-Core25 complex.

### Recombinant Protein Expression

Synthetic expression plasmids (GenScript) encoding HB3-Core25 (pET-29b­(+)) and the GST-tagged variant (pGEX-4T-1) were transformed into *Escherichia coli* BL21 Star (Invitrogen). SARS-CoV-2 M^pro^ sequence was cloned into the same GST vector. Cultures were grown in a Luria–Bertani (LB) medium supplemented with 50 mg/mL ampicillin or 10 mg/mL kanamycin. At OD_600 nm_ = 0.5–0.8, protein expression was induced with 1 mM isopropyl β-d-1-thiogalactopyranoside (IPTG) at 22 °C, 225 rpm, for 20 h. Cells were pelleted (7000 g, 4 °C, for 30 min) and resuspended in lysis buffer (50 mM Tris-HCl pH 8.0, 500 mM NaCl, 20 mM imidazole) supplemented with protease inhibitor (Roche).

### Affinity Purification

Bacterial cells were disrupted by sonication (Vibracell VCX 750 Sonicator) on ice, and lysates were purified via metal ion affinity chromatography using a HisTrap HP column (Cytiva). Proteins were eluted with an imidazole gradient (up to 500 mM) in elution buffer (50 mM Tris-HCl, pH 8.0, 500 mM NaCl_2_) under a stepwise fashion. For SARS-CoV-2 M^pro^ purification, 10 mM dithiothreitol (DTT) was added to all of the buffers. The GST-tagged version of the miniprotein (GST-HB-Core25) was purified using a GSTrap FF column (Cytiva) through isocratic elution in 50 mM Tris-HCl, pH 8.0, 10 mM reduced glutathione. SDS-PAGE assessed purity (BlueSafe stain (NZYtech)), and the protein concentration was determined by spectrophotometry (Nanodrop Onec, Thermo) using extinction coefficients at 280 nm (M^pro^) or 227 nm (HB3-Core25).

### Size-Exclusion Chromatography (SEC)

Proteins were dialyzed overnight in 1× phosphate-buffered saline (PBS) at 4 °C and concentrated using Amicon Ultra 15 filters (Millipore). HB3-Core25 (100 μM) was incubated with M^pro^ (30 μM) at 22 °C for 30 min to form complexes. The resulting complexes were loaded onto a HiLoad 26/600 Superdex 75 pg column (Cytiva). Peaks were assigned by comparison and data interpolation to a standard curve. Partition coefficients (*K*_av_) were calculated for protein complex characterization.

### Circular Dichroism (CD) Spectroscopy

The secondary structure was analyzed on a J-1100 spectropolarimeter (Jasco) from 190 to 260 nm at 25 °C, with three accumulations and a scanning speed of 20 nm/min, using 1 mm quartz cuvettes. HB3-core25 (5 μM) and M^pro^ (10 μM) were diluted in 100 mM sodium phosphate buffer (pH 7.4). Thermal stability was assessed by monitoring ellipticity at 222 nm from 10 to 100 °C in 2 °C increments and 30 s equilibration steps, while reversibility of unfolding was assessed by cooling the same sample from 100 to 10 °C. Complex melting temperature (*T*_m_) was determined after incubation of equimolar protein conditions (5 μM, 22 °C, 30 min) during thermal titration (10–100 °C) and compared to the denaturation curve of M^pro^ alone at the same concentration (5 μM). All experiments were repeated three times.

### Microscale Thermophoresis (MST)

The equilibrium dissociation constant (*K*_D_) of the M^pro^-HB3-Core25 interaction was measured by MST using fluorescently labeled M^pro^ (RED-MALEIMIDE second Generation protein labeling kit, NanoTemper). Labeled M^pro^ (250 nM) was titrated with 16 serial dilutions of GST-HB3-Core25 (101–0.00308 μM). Following incubation (22 °C, 30 min, in the dark), the samples were loaded into standard MST capillaries and analyzed on a Monolith NT.115 instrument (NanoTemper). The *K*_D_ values, along with noise and user-estimated errors, were calculated using the MO-affinity Analysis software.

### SARS-CoV-2 M^pro^ Enzymatic Inhibition Assay

The inhibitory effect of HB3-Core25 on M^pro^ was assessed using a luminogenic substrate (Z-RLRGG-aminoluciferin, Promega), which mimics the N-terminal protease’s autocleavage sequence. The assay was conducted in 96-well plates with 40 μM substrate, 0.16 μg/μL M^pro^, and serial dilutions of HB3-Core25 (1 nM–25 μM), incubated at 37 °C for 1 h. Luminescence was measured after adding 50 μL of detection reagent (Promega) to each well and a 20 min stabilization period using a Glomax luminometer (Promega). The results were compared to the positive control (M^pro^ in the absence of the inhibitor). Mean inhibitory concentrations (IC_50_) were calculated from dose–response curves using GraphPad Prism 6.

## Results

### *De Novo* M^pro^ Binding Protein Folds into the Desired Conformation and Exhibits High Stability in Solution

The N-terminal region of M^pro^ from both SARS-CoV-1 and SARS-Cov-2 has previously been shown to inhibit proteolytic activity by disrupting homodimer formation, which is essential for protease function.^9,42^ To identify the key residues contributing to binding affinity in the M^pro^-N8 complex, we first performed alanine scanning (AS) mutagenesis across the peptide sequence. This analysis revealed that substitutions R4A and M6A led to significant increases in the binding free energy (Figure S1), indicating that R4 mediates critical electrostatic interactions, particularly with E290 of M^pro^, while M6 is essential for hydrophobic packing within the complex. These findings identified the R4 and M6 residues as key for rational inhibitor design.

Guided by this finding, we pursued a *de novo* design strategy to engineer a stable miniprotein inhibitor targeting M^pro^. We began by grafting the key residues R4 and M6 onto a 16-residue α-helix, which served as the central motif of a three-helix bundle topology (Figure 1A). A total of 45 000 simulations were conducted to generate candidate scaffolds. Designs were filtered based on their ability to form well-packet hydrophobic cores, avoid steric clashes with M^pro^, and incorporate loops as short as possible to enhance rigidity. These criteria ensured optimal side chain packing around the central helix, which housed the transplanted binding residues.

Following scaffold selection, we carried out extensive sequence design using Rosetta’s FastRelax protocol, generating 50 000 sequences optimized for structural compatibility and target binding. Three top candidates were initially selected based on the following metrics: total energy per residue (ranging from −3.53 to −3.41 kcal/mol), buried nonpolar surface area (nSPA, 60.72–68.93 Å^2^), and overall packing efficiency. However, only one exhibited the desired biological activity experimentally. For conciseness, we will only discuss this single candidate (Figure S2 and Table S1). Importantly, the designed interfaces showed improved predicted binding affinity (ΔΔ*G*) compared to the original N8 peptide, along with strong shape complementarity (Sc > 0.66), excellent internal packing (Packstat >0.66), and a high contact molecular surface (CMS > 498). A comparison of the designed sequence with the N8 peptide reveals a significant sequence difference, where only 3 out 8 residues are identical. Besides the grafted R4 and M6, the miniprotein has an alanine at the A7 corresponding position in the N8 peptide. (The N8 peptide sequence is SGFRKMAF, while the corresponding region in the designed protein is HEARVMAM.)

To ensure that the designed sequence would indeed adopt the intended fold, we performed structural validation using both *ab initio* relax folding simulations and AlphaFold2-based predictions. Energy landscape analysis revealed a narrow and well-defined funnel, consistent with a strongly preferred global minimum and reduced conformational heterogeneity. The RMSD between the designed model and the lowest-energy folded structure was 0.0 Å (Figure 1B), suggesting a perfect structural agreement. Similarly, AlphaFold2 and AlphaFold3 predictions yielded high per-residue confidence scores, with per-residue measure of local confidence (pLDDT) values above 95%, reinforcing the reliability of the generated models. The predicted structure of the complex also exhibited remarkable agreement with the AlphaFold2 and AlphaFold3 models, with backbone RMSD values of ca. 0.6 Å further validating the reliability of the model. Altogether, these results demonstrate that the designed miniprotein adopts the desired three-helix bundle with high fidelity and stability, making it a promising scaffold for M^pro^ inhibition.

### M^pro^-HB3-Core25 Complex Forms a Stable Association in Solution

While Rosetta implicitly accounts for solvent effects during interface design, penalizing polar atoms with unsatisfied hydrogen bonds, explicit solvent dynamics is essential for accurately evaluating the stability of protein–protein interactions. An MD simulation of the M^pro^-HB3-Core25 system was carried out to refine the key interactions of the structural complex. A simulation of the native M^pro^ homodimer was also performed for comparison purposes. Simulations were conducted at 25 °C, over a 1 μs trajectory. Both complexes remained structurally stable throughout the simulation time, exhibiting low RMSD fluctuations in the backbone atoms across all chains (Figures 2A,2B and S3). The center-of-mass (COM) distances between binding partners also remained consistent, and fluctuations in the RMSD of interfacial heavy atoms showed minimal RMSD variations, indicating the structural integrity of the interfaces (Figure 2A,B).

**Figure 2:**
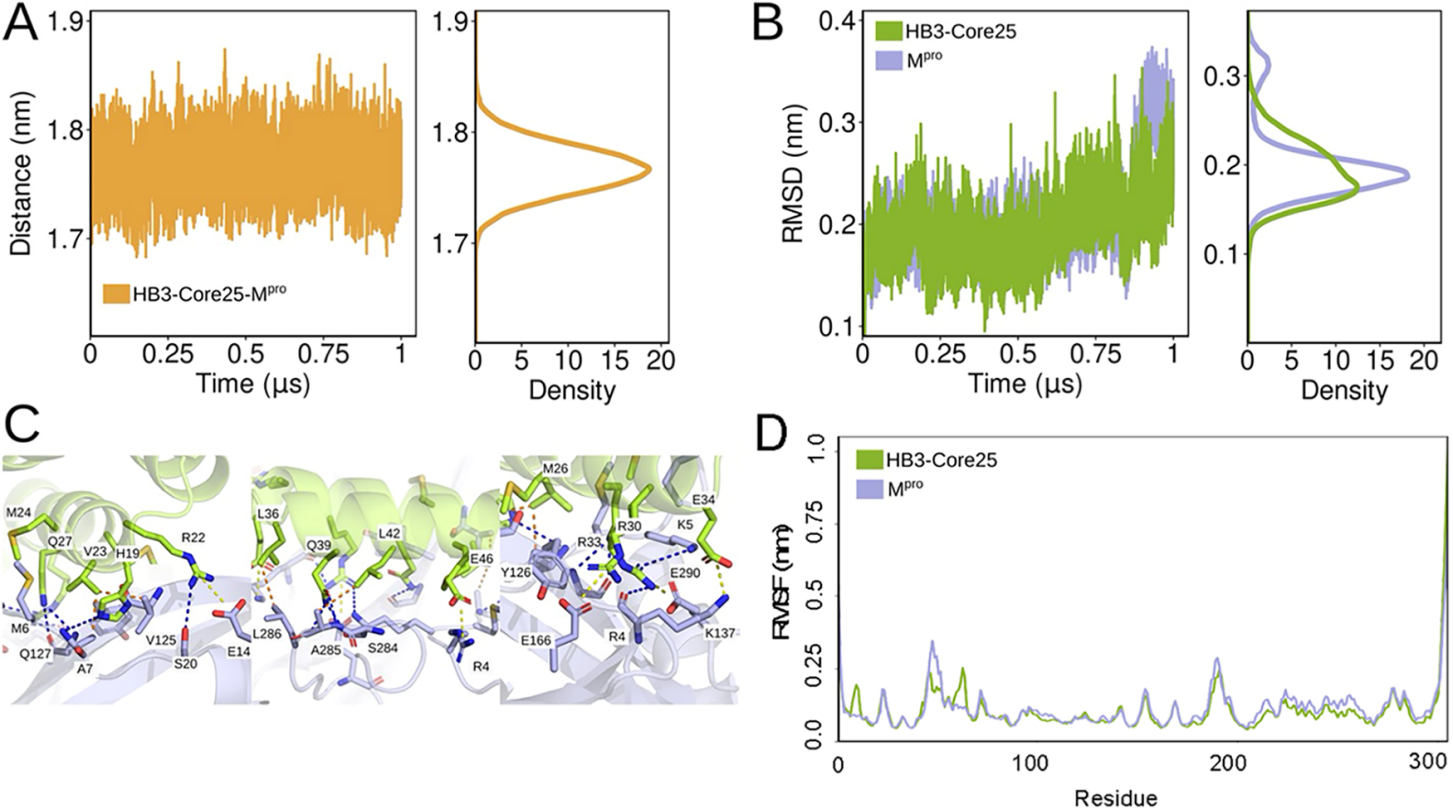
Structural dynamics and key interactions in the M^pro^ dimer and M^pro^-HB3-Core25. (A) Distance between the centers of mass of the heavy atoms at the interface residues. (B) RMSD of the heavy chain atoms at the interface residues of M^pro^ (blue) and HB3-Core25 (green). (C) Protein–protein interface interaction represented by residue distances in Å. The M^pro^ interface is colored blue, and HB3-Core25 is shown in green. Dashed lines indicate possible interactions between the molecules: hydrogen bonds (orange), salt bridges (purple), and hydrophobic interactions (orange). Interface was defined as any atomic contacts that fell within a 6 Å distance. (D) RMSF of residue-averaged heavy chain atoms of M^pro^ in the presence (green) and absence of the miniprotein (blue). (The M^pro^ curve is shown as the resulting average of the two homodimer chains).

To understand the molecular basis of this stability, we characterized the key interfacial interactions maintained during the simulations. The M^pro^-HB3-Core25 interface was primarily stabilized by hydrogen bonds and salt bridges, formed by residue pairs including R30A–E290B (100%), M26A–Y126B (99.7%), Q27A–K5B (97.5%), L36A–L286B (96%), E34A–K189B (93.4%), M24A–58B (90.6%), and E46A–R4B (89.5%) (Figure 2C). These interactions were present for the majority of the simulation time and closely mirrored those observed in the native M^pro^ homodimer (Table S2). Although hydrophobic contacts constitute a smaller portion of the total interactions, they remained stable over the course of the simulation, contributing additional support to the interface integrity. The overall dynamical fluctuation remained unchanged for M^pro^ upon miniprotein binding (Figure 2D). A very low RMSF is observed in both cases throughout the protein except for the C-terminal residues in both cases. The complex was further validated by calculating the binding affinity of the miniprotein to M^pro^ by PBEE,^43^ a recently developed ML-based software to estimate protein–protein binding free energies with unprecedent accuracy. PBEE predicts a Δ*G*_bind_ of −10.72 kcal/mol, which corresponds to a *K*_D_ of ca. 10^-8^ M. Collectively, these results suggest that the protein–protein interactions engineered by Rosetta form a robust and persistent interface under physiologically relevant conditions. These results suggest that HB3-Core25 exhibits sufficient structural and thermodynamic stability to engage M^pro^ in a dimer-blocking mode.

### Miniproteins Are Thermostable and Maintain the Desired Conformation

To validate the *in silico* predictions and evaluate the biophysical properties of HB3-Core25, the miniprotein was recombinantly expressed in *E. coli*. Expression was successful and yielded 0.9 g/L (Figure S4A). The protein remained highly soluble throughout purification and handling, as evidenced by the absence of aggregation or viscosity changes. To assess whether the designed secondary structure was preserved after expression, we performed circular dichroism (CD) spectroscopy. The CD spectrum displayed hallmark features of α-helical structures, with characteristic minima at 208 and 222 nm, and a maximum at 193 nm (Figure 3A), confirming that HB3-Core25 adopts an α-helical conformation in solution. Moreover, the CD signal remained unchanged upon heating, indicating resistance to complete thermal denaturation. These findings are consistent with the MD simulations conducted at comparable temperatures (Figure 1D), further supporting the structural robustness of the miniprotein.

**Figure 3:**
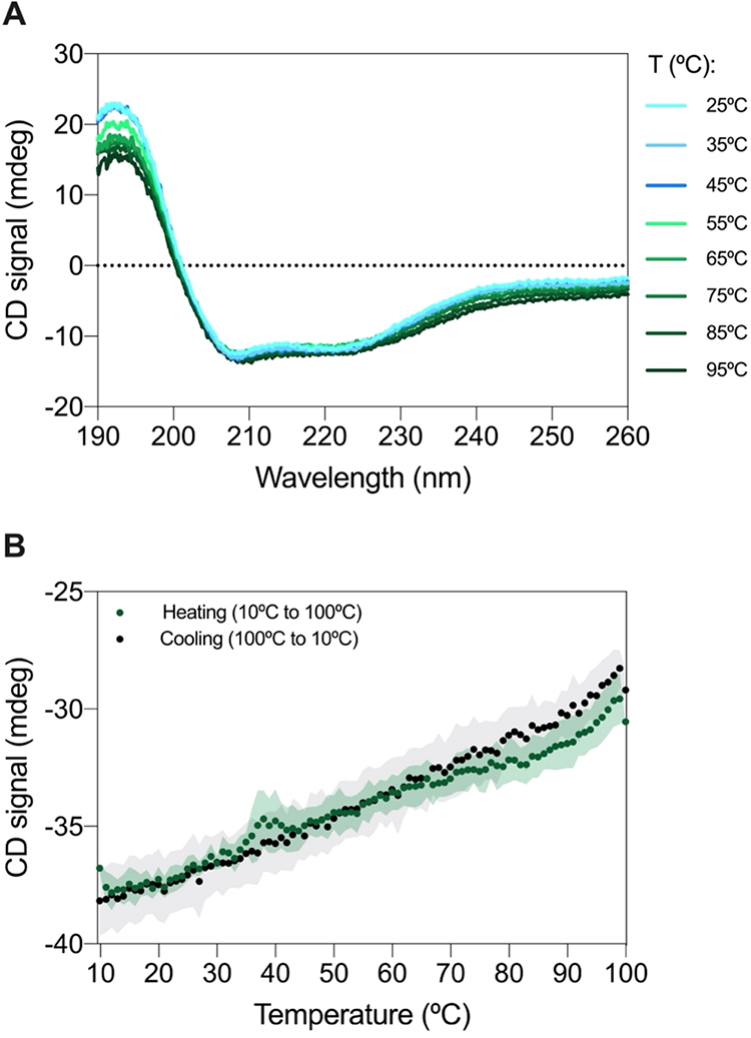
Biophysical characterization of the HB3-Core25 miniprotein. (A) CD spectra of HB3-Core25 recorded over the wavelength range of 190–260 nm from 25 to 95 °C, at 10 °C intervals. (B) CD spectra of HB3-Core25 at a fixed wavelength of 222 nm during thermal titration from 10 to 100 °C (green line) and from 100 to 10 °C (black line). Shaded areas represent experimental error over three independent measurements.

To evaluate thermal stability more precisely, we conducted thermal denaturation and refolding capacity assays by monitoring the CD signal at 222 nm across a temperature range of 10–100 °C (upon sample heating and cooling). Remarkably, no significant structural changes were detected within this range, suggesting that the miniprotein remains fully folded, even at the highest tested temperature (Figure 3B). This result indicates a melting temperature (*T*_m_) exceeding 100 °C and highlights the exceptional thermostability of HB3-Core25. Noteworthy, the heating and cooling CD spectra showed an overlapping pattern that indicates not only the reversibility of unfolding but also that HB3-Core25 exhibits a robust folding pathway, as expected from a well-designed miniprotein.

### HB3-Core25 Miniprotein Forms a Complex with SARS-CoV-2 M^pro^ and Inhibits Its Catalytic Activity

Although the HB3-Core25 miniprotein was shown to be structurally robust, its inhibitory potential against M^pro^ depends on its ability to form a functional complex with the protease. CD spectroscopy and microscale thermophoresis experiments were performed to characterize the interaction between the recombinantly produced SARS-CoV-2 M^pro^ (Figure S4C) and HB3-Core25. CD spectroscopy of the recombinant SARS-CoV-2 M^pro^ confirmed that the protease possesses a mixed secondary structure, displaying characteristic minima at 208 and 225 nm, and a maximum at 195 nm, consistent with both α-helical and β-sheet elements (Figure S5). Thermal denaturation assays demonstrated a complete loss of secondary structure above 55 °C, with a *T*_m_ of 46.8 °C (Figure 4A).

**Figure 4:**
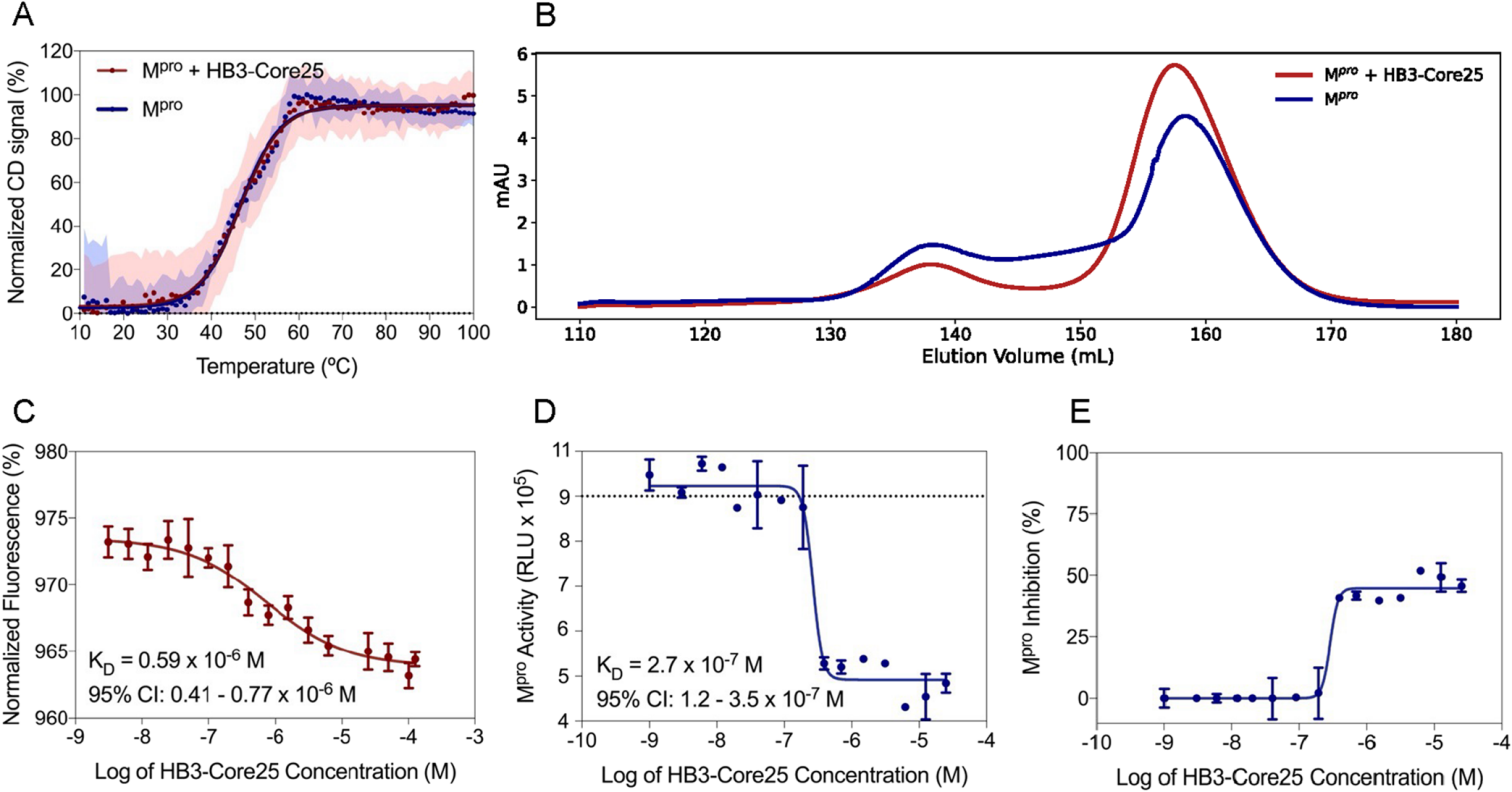
Analysis of M^pro^ binding and inhibition by HB3-Core25. (A) CD spectra of SARS-CoV-2 M^pro^ (blue line and dots) and the M^pro^- HB3-Core25 complex (red line and dots) at a fixed wavelength of 222 nm, during thermal titration from 10 to 100 °C. The Tm of SARS-CoV-2 M^pro^ is 46.8 °C and it was kept unchanged upon binding to the inhibitor. M^pro^ concentration was kept constant at 5 μM for both experiments. Shaded areas represent experimental error over three independent measurements. (B) Size-exclusion chromatography of M^pro^ in the presence and absence of the HB3-Core25 miniprotein. The chromatogram shows two broad peaks with maxima values centered at 138 and 158 elution volumes. The first peak corresponds to the dimer population, while the second peak corresponds to the monomeric M^pro^. A comparison shows a relatively larger population of the monomeric form of M^pro^ in the presence of the miniprotein. Integration of the area under the curve of the second peak reveals a 40% increase in the area for the complex. (C) MST binding assays. Each point on the dotted line represents the change in fluorescence of the labeled M^pro^ as the concentration of the HB3-Core25 inhibitor increases. The dissociation constant K_D_ for the binding of the HB3-Core25 miniproteins to M^pro^ is 0.59 μM, 95% CI: 0.41–0.77 μM (dashed line). (D) M^pro^ enzymatic inhibition assay with HB3-Core25. Each point (blue) on the curve represents the concentration of the inhibitor. The inhibitory concentration (IC50) of HB3-Core25 corresponds to 0.27 μM, 95% CI: 0.12–0.35 μM. RLU stands for relative light units. The gray dotted line indicates the luminescence signal of the positive control. (E) Percentage of inhibition of M^pro^ enzymatic activity with increasing concentrations of HB3-Core25.

To determine whether HB3-Core25 forms a stable complex with M^pro^, thermal shift assays were performed. Complex formation typically results in increased or unchanged thermostability due to cooperative folding and interaction.^35^ Upon incubation with HB3-Core25, the Tm of M^pro^ remained unaltered, indicating the formation of a thermodynamically stable complex and absence of structural changes in M^pro^ upon binding (Figure 4A). It is important to note that due to the monomer–dimer equilibrium of M^pro^, the Tm is concentration-dependent. To ensure comparability, all denaturation assays were conducted using the same M^pro^ concentration. Binding affinity was further quantified by microscale thermophoresis (MST), using a fluorophore-labeled M^pro^ and a GST-tagged HB3-Core25 construct to enhance molecular weight and signal intensity (Figure S4B). The assays yielded a dissociation constant (*K*_D_) of 5.9 × 10^–7^ M, 95% confidence interval (CI): (4.1–0.7) × 10^–7^ M, confirming a direct and specific interaction between the miniprotein and M^pro^ (Figure 4B). The observed fluorescence changes were dose-dependent, further validating the binding interaction. Moreover, to confirm HB3-Core25 is effectively binding to the M^pro^ dimerization interface, MST assays were performed using an Mpro variant harboring two mutations previously demonstrated to abrogate dimerization (E290A and R298A)^44^ (Figure S6). While HB3-Core25 binds to wild-type M^pro^, no binding was detected with the dimerization-deficient mutant, supporting our conclusion that HB3-Core25 specifically targets the dimerization interface of M^pro^.

Finally, to assess the functional consequence of this interaction, enzymatic inhibition tests were conducted. HB3-Core25 effectively suppressed the catalytic activity of M^pro^, with an inhibitory concentration (IC_50_) of 0.27 μM, 95% CI: 0.12–0.35 μM. At a concentration of 25 μM, the miniprotein inhibited 51.1% of substrate cleavage (Figure 4C,D). These results demonstrate that HB3-Core25 functions as an M^pro^ inhibitor, likely by interfering with the dimerization process required for enzymatic activation. It is supported by SEC experiments, where HB3-Core25 is shown to increase the M^pro^ monomer population by around 40% (Figure 4B).

## Discussion

Viral proteases, such as the main protease (M^pro^) of SARS-CoV-2, are attractive drug targets due to their essential role in viral replication and their high degree of conservation across coronavirus strains.^42,45–47^ Since the functional unit of M^pro^ is a homodimer, blockers of protein dimerization should be in principle able to diminish its catalytic activity and may lead to potential broad-spectrum antiviral drugs. In this regard, eight small molecules targeting the M^pro^ dimerization region have been developed by other study groups to inhibit M^pro^ protease activity.^47–50^ While effective to some extent, three of those molecules have shown no detectable half-maximal effective concentration (EC_50_) or half-maximal inhibitory concentration (IC_50_). The other class of inhibitors comprises peptides with high selectivity for M^pro^ and with low accumulation levels in the *in vivo* studies.^51^ Among these peptides, Ding et al. have described that the eight N-terminal residues (SGFRKMAF, named N8) of M^pro^ act as a dimerization inhibitor.^13,14^ This strategy, along with the presence of well-defined M^pro^ allosteric sites, has been largely explored.^48^

These studies highlighted that the N-terminus of M^pro^ plays a critical role in mediating dimerization.^4,8^ Specifically, N-terminal residues S1, R4, and M6 are essential for stabilizing the dimer interface. S1 forms key interactions with F140 and E166 of the adjacent monomer, which are vital for maintaining the proper conformation of the catalytic site.^52,53^ Additionally, R4 establishes a salt bridge with E290 from the opposite subunit, further contributing to dimer stability.^13,53–55^ M6, in turn, projects into a hydrophobic pocket of the neighboring protomer, reinforcing the dimer through hydrophobic interactions.^13^ To effectively promote both M^pro^ dimer dissociation and inhibition of its catalytic activity, a molecule is required to form, at minimum, a hydrogen bond with E166.^56^ The critical role of this residue (conserved across all known human coronaviruses) has been highlighted in recent studies as essential for effective inhibition.^57^ Notably, Goyal and Goyal have identified E166 as one of the key residues that should be targeted to disrupt SARS-CoV M^pro^ dimerization.^4^

In this context, we computationally designed HB3-Core25, a *de novo* miniprotein aimed to incorporate key traits of the binding interface of N8 and enhance interaction with the M^pro^ dimerization interface to inhibit its enzymatic activity. This strategy was motivated by the fact that, when compared to linear peptides, small proteins have greater functional diversity and higher structural stability through the precise design of noncovalent interactions, which results in superior biological function.^18,58–60^ On the other hand, a key challenge in computational protein design has historically been the low rate of experimental success—approximately 2% at the time this study was initiated.^61,62^ However, recent advances in artificial intelligence and deep learning-guided modeling have dramatically increased success rates to 30% or more.^63,64^ Furthermore, when the design process incorporates chemical intuition in the manual supervision of interface geometry and residue–residue interactions, success rates can approach 50%.^65^ It is important to highlight that modern computational design strategies enable the prediction of high-confidence protein candidates.^62,66^ Rather than requiring large-scale experimental screening, these approaches prioritize variants with the greatest likelihood of correct folding and target engagement. Previous studies have demonstrated that even a single rationally designed miniprotein can effectively validate the intended mechanism of action, particularly when guided by strong biophysical principles and structural intuition.^66,67^ The development of HB3-Core25 reflects this approach. Among several computational candidates, HB3-Core25 was selected not only based on predicted stability and binding energy but also guided by visual inspection and chemically informed assessment of its interface geometry. Other studies have shown that conformational stabilization of miniproteins by substantial engineering is crucial to achieve potent inhibitory activity.^68–70^ Adding modifications to the loops that connect the α-helices and redesigning new interactions to stabilize the coiled-coil structures are required to strongly enhance miniprotein’s propensity to fold into the intended compact three-helix bundle and to exert its proper biological function.

Validating this approach, the rational design of HB3-Core25 resulted in a miniprotein exhibiting a melting temperature exceeding 100 °C and a 24-fold higher affinity (*K*_D_ = 0.59 μM, CI: 0.41–0.77 μM) by M^pro^ than the original N8 peptide used as a template (*K*_D_ ≈ 14 μM).^13^ In addition, the HB3-Core25 miniprotein decreased M^pro^ *in vitro* substrate cleavage in half, with an IC_50_ of 0.27 μM, CI: 0.12–0.35 μM, about 2000 times less than the amount required of the N8 peptide to inhibit 46% of M^pro^’s catalytic activity. When HB3-Core25 is compared with previously reported peptide inhibitors of M^pro^, its inhibitory profile appears consistent with or superior to those targeting either the dimerization interface or the catalytic site. Stewart et al.^12^ reported peptide inhibitors optimized for M^pro^ dimer disruption, with affinities in the micromolar range but no evidence of thermal stability or structural rigidity comparable to *de novo* miniproteins. Moreover, peptides targeting the catalytic site, such as those described by Johansen-Leete et al. and macrocyclic peptides described by Harrison et al.,^10,11^ achieved IC50 values in the low micromolar range but showed limited stability in biochemical assays, posing a challenge for future *in vivo* applications. While other catalytic site inhibitors (such as dronedarone)^71^ can act directly at the catalytic site with reported potencies in the low micromolar to nanomolar range, inhibitors targeting M^pro^ dimerization interface typically require higher concentrations due to the broader and defined interaction surface. However, this strategy offers the advantage of acting on a highly conserved region of M^pro^, which may reduce the likelihood of resistance development.

While the binding affinity of HB3-Core25 to M^pro^ is comparable to the well-characterized GC376, a prodrug (*K*_D_ = 0.15 ± 0.03 μM), the clinically approved inhibitor nirmatrelvir displays a higher affinity (NMR/r; *K*_D_ = 0.007 ± 0.004 μM).^72^ It is worth noting that these two compounds target M^pro^’s active site, instead of the enzyme’s dimer interface, where high binding affinity does not seem to be a strict requirement for inhibitory activity.^73^ Silvestrini et al. investigated a range of compounds that inhibit M^pro^’s catalytic activity by targeting either the monomer–monomer interface or the active site.^56^ Their results suggest a synergistic mechanism where compounds that do not strongly disrupt dimerization can still effectively inhibit enzymatic activity by acting on both sites. In line with these findings, HB3-Core25 miniprotein decreased M^pro^ *in vitro* substrate cleavage in half, even though the dissociation constant was also in the submicromolar range. This result can be partially explained by the fact that M^pro^ exists in a dynamic dimer–monomer equilibrium with dissociation constants reported between 1 and 7 μM.^4,42,57,72,74^ Although this indicates a relatively weak affinity in absolute terms compared with nanomolar binders, it does not necessarily mean ineffective dimerization. Biologically, the intracellular concentration of M^pro^ favors dimer formation despite this, and therefore M^pro^ predominantly exists in its dimeric and catalytic active state. Therefore, at typical experimental concentrations, and even when a compound perturbs this equilibrium, a fraction of M^pro^ remains in its dimeric, active form and can still perform enzymatic activity in the presence of the substrate.

Altogether, our results demonstrate that HB3-Core25 is a thermostable inhibitor of SARS-CoV-2 M^pro^. Designed to target the dimerization interface intracellularly, HB3-Core25 impairs M^pro^ activity likely by interfering in dimer formation (Figure 4B), offering an alternative mechanism of action to active-site inhibitors. In addition, it can also be synergistically used with M^pro^’s active-site inhibitors to achieve higher levels of inhibition of the enzyme’s catalytic activity. While the current study focuses on the design and biophysical characterization of the miniprotein, immunogenicity and proteolytic stability analysis as well as strategies covering conjugation to cell-penetrating peptides, nanoparticle encapsulation, or mRNA-based delivery could be explored in future studies to ensure intracellular delivery, biocompatibility, and safety. Future *in vitro* and *in vivo* antiviral activity testing will be crucial to translate these findings into clinical applications. Nonetheless, this work underscores that computationally designed molecules, particularly when coupled with chemical intuition, can produce functional inhibitors with therapeutic potential.

## Supplementary Material



## Data Availability

Data supporting the findings of this study, including molecular data in PDB format and simulation scripts, are available at https://github.com/rlinslab/miniprotein-mpro-data. The Rosetta software is available at https://rosettacommons.org, GROMACS is available at https://www.gromacs.org, the PBEE software is available at https://github.com/chavesejf/PBEE, and Plumed is available at https://www.plumed.org.

## Notes

HB3-Core25 protein sequence and interaction profile between the N8 peptide and M^pro^ for SARS-CoV-2; metrics of monomers (A) and interfaces (B) for the selected sequences; structural stability analysis of protein–protein complexes via root-mean square deviation (RMSD); production of SARS-CoV-2 M^pro^ and HB3_CORE 25 recombinant proteins; biophysical characterization of SARS-CoV-2 M^pro^; MST binding assay of double mutant E290A/R298A monomeric M^pro^; metrics of the monomers and the interfaces between the miniproteins and M^pro^, as well as the known dimerization inhibitors of M^pro^ from SARS-CoV-2; and percentages of interactions between amino acid residues in protein–protein complexes during molecular dynamics simulations (PDF)

## Abbreviations

AS: alanine scanning
CD: circular dichroism
CMS: contact molecular surface
COM: center of mass
ΔInsatLH: buried unsatisfied hydrogen bonds
FES: free energy surface
HCV: hepatitis C virus
HIV: human immunodeficiency virus
IC_50_: inhibitory concentration
IPTG: isopropyl β-d-1-thiogalactopyranoside
*K*_D_: dissociation constant
LB: Luria–Bertani
MD: molecular dynamics
M^pro^: main chymotrypsin-like protease
ML: machine learning
MST: microscale thermophoresis
NSP: nonstructural protein
nspa: buried nonpolar surface area
PL^pro^: papain-like protease
pLDDT: per-residue measure of local confidence
RMSD: root-mean-square deviation
RMSF: root-mean-square fluctuation
SARS-CoV-2: severe acute respiratory syndrome coronavirus 2
SASA: buried solvent-accessible surface area
SEC: size-exclusion chromatography
SS-Sc: shape complementarity
*T*_m_: melting temperature
