# Supplementary material for: De Novo-Designed Miniprotein Inhibits the Enzymatic Activity of the SARS-CoV‑2 Main Protease
Source: J Chem Inf Model. 2025 Oct 8;65(20):11314–25. doi: 10.1021/acs.jcim.5c01708 (PMC12570131; doi:10.1021/acs.jcim.5c01708)
Supplement: Supplementary file 1 [file ci5c01708_si_001.pdf]

SUPPORTING INFORMATION

De novo designed miniprotein inhibits the enzymatic activity of the SARS-CoV-2 main protease

Tayná E. Lima[1,δ]; Emerson G. Moreira[1,2,δ]; Danilo F. Coêlho[2], Carlos H. B. Cruz[3]; Rafael Dhalia[1]; Bruno H. S. Leite[1]; Lícya S. Xavier[1]; Marta Perez Illana[1]; Gabriel L. Wallau[4,5]; Isabelle F.T. Viana[1,2,5,6,*]; Roberto D. Lins[1,5*].

[1]Department of Virology, Aggeu Magalhães Institute, Oswaldo Cruz Foundation, Recife, 50740-465, Brazil.

[2]Department of Fundamental Chemistry, Federal University of Pernambuco, Recife, 50740-540, Brazil.

[3]Institute of Structural and Molecular Biology, University College London, London, WC1E 7HX, United Kingdom.

[4]Department of Entomology, Aggeu Magalhães Institute, Oswaldo Cruz Foundation, Recife, 50740-465, Brazil.

[5]Genomic Network Fiocruz, Oswaldo Cruz Foundation, Recife, 50740-465, Brazil.

[6]Graduate Program in Materials Science, Federal University of Pernambuco, Recife, 50740-540, Brazil.

[δ] These authors share first authorship.

*** Corresponding Authors**

Roberto D. Lins – Department of Virology, Aggeu Magalhães Institute, Oswaldo Cruz Foundation, Recife, 50740-465, Brazil. Email: roberto.neto@fiocruz.br (ORCID 0000-0002-3983-8025).

Isabelle F. T. Viana – Department of Virology, Aggeu Magalhães Institute, Oswaldo Cruz Foundation, Recife, 50740-465, Brazil. Email: isabelle.viana@fiocruz.br (ORCID 0000-0003-4648-6635)

Supplementary content

Figure S1. HB3-Core25 protein sequence and interaction profile between the N8 peptide and M^{pro} for SARS-CoV-2.

Figure S2. Metrics of monomers (A) and interfaces (B) for the selected sequences.

Figure S3. Structural Stability Analysis of Protein-Protein Complexes via Root Mean Square Deviation (RMSD).

Figure S4. Production of SARS-CoV-2 M^{pro} and HB3_CORE 25 recombinant proteins.

Figure S5. Biophysical Characterization of SARS-CoV-2 M^{pro}.

Figure S6. MST binding assay of double mutant E290A / R298A monomeric M^{pro}.

Table S1. Metrics of the monomers and the interfaces between the mini-proteins and M^{pro}, as well as the known dimerization inhibitors of the M^{pro} from SARS-CoV-2.

Table S2. Percentages of interactions between amino acid residues in protein-protein complexes during molecular dynamics simulations.

Rosetta scripts.

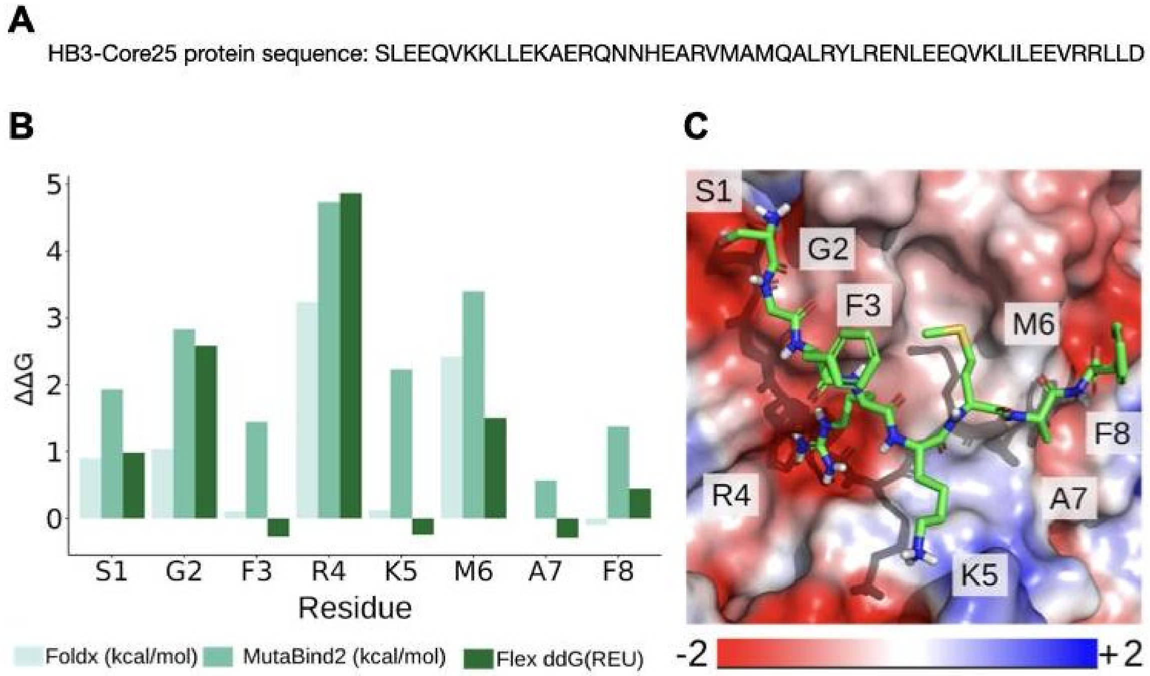

Figure S1. HB3-Core25 protein sequence and interaction profile between the N8 peptide and M^pro^ for SARS-CoV-2. A) HB3-Core25 protein sequence. B) ΔΔG for alanine scanning. ΔΔG values are represented in shades of green: light green for FoldX, green for MutaBind2, and dark green for Flex ddG (Rosetta). C) Electrostatic surface of the interaction between the N8 peptide and Mpro for SARS-CoV-2.

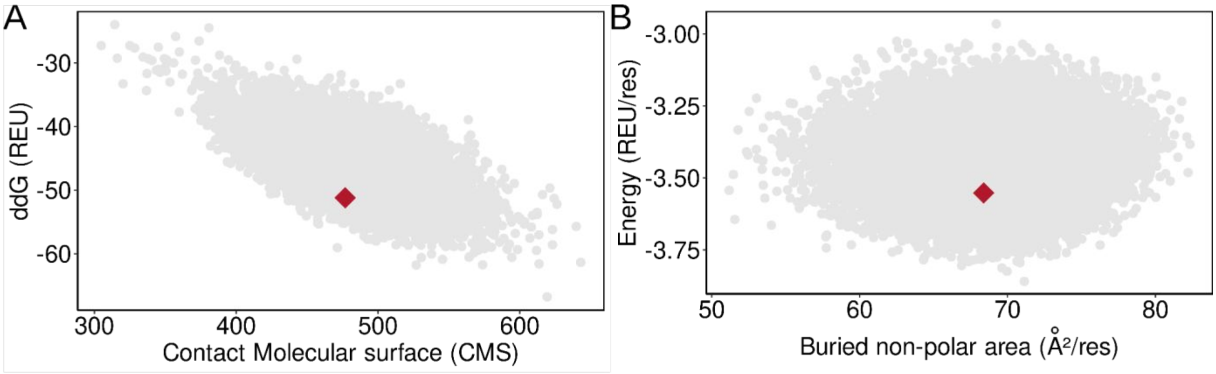

Figure S2. Metrics of monomers (A) and interfaces (B) for the selected sequences. In gray, all designed sequences are shown, while the red diamond represents the selected HB3-Core25 sequence.

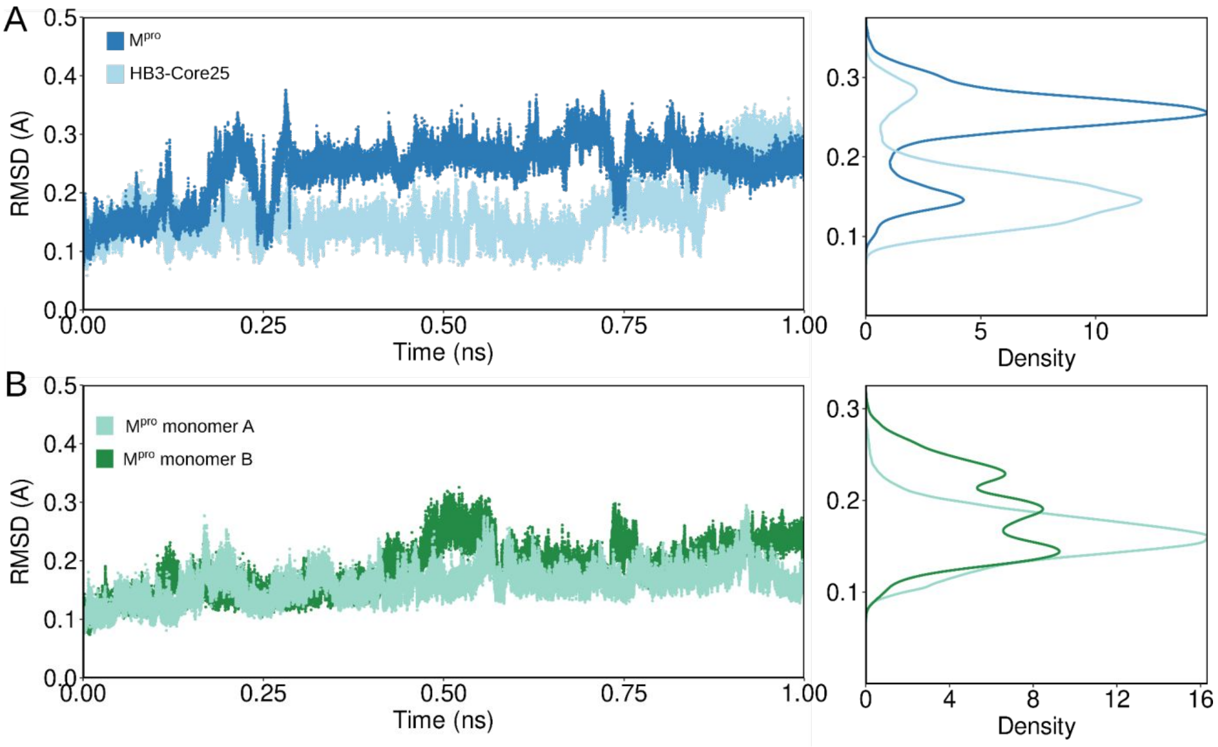

Figure S3. Structural Stability Analysis of Protein-Protein Complexes via Root Mean Square Deviation (RMSD). A) RMSD of main chain atoms over time for the complexes: M^{pro}-HB3-Core25 (blue) and M^{pro} (blue dark). B) RMSD of main chain atoms over time for the M^{pro} homodimer.

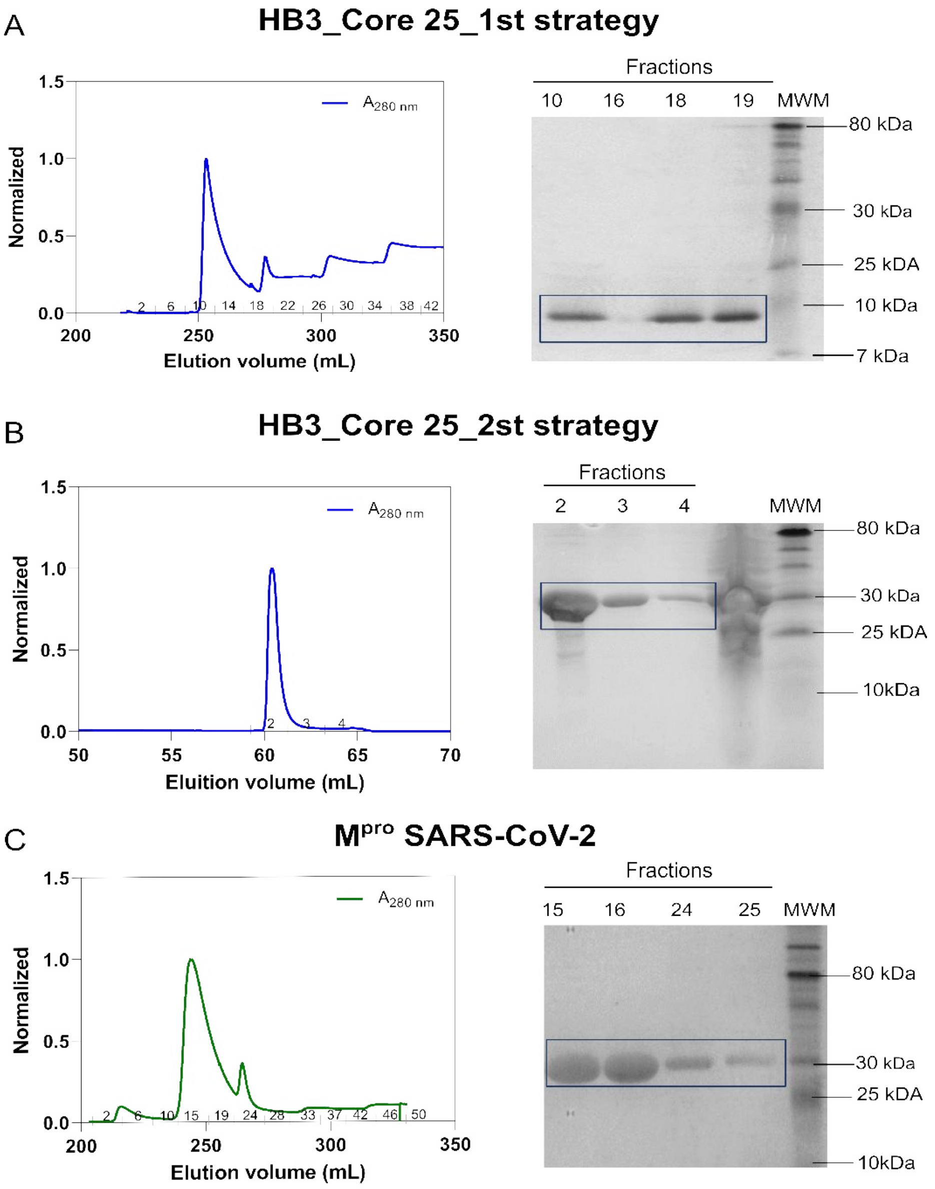

Figure S4. Production of SARS-CoV-2 M^{pro} and HB3_CORE 25 recombinant proteins. (A) Chromatogram resulting from metal ion affinity purification of the HB3_CORE 25 miniprotein (left). Gel resulting from sodium dodecyl sulfate-polyacrylamide gel electrophoresis run (SDS-PAGE) was carried out with fractions 10, 16, 18 and 19 eluted from purification (8,79 kDa / right). (B) Chromatogram resulting from glutathione-s-transferase (GST) affinity purification of the HB3_CORE 25 miniprotein (left). Gel resulting from sodium dodecyl sulfate-polyacrylamide gel electrophoresis run (SDS-PAGE) was carried out with fractions 02, 03, and 04 eluted from purification (35,29 KDa / right). (C) Chromatogram resulting from metal ion affinity purification of the SARS-CoV-2 M^{pro} protein (left). Gel resulting from sodium dodecyl sulfate-polyacrylamide gel electrophoresis run (SDS-PAGE) was carried out with fractions 15,

16, 24 and 25 eluted from purification (35,59 / right). The molecular weight marker (MWM) used was the Prestained Protein Marker Broad Range (Cell Signaling Technology®).

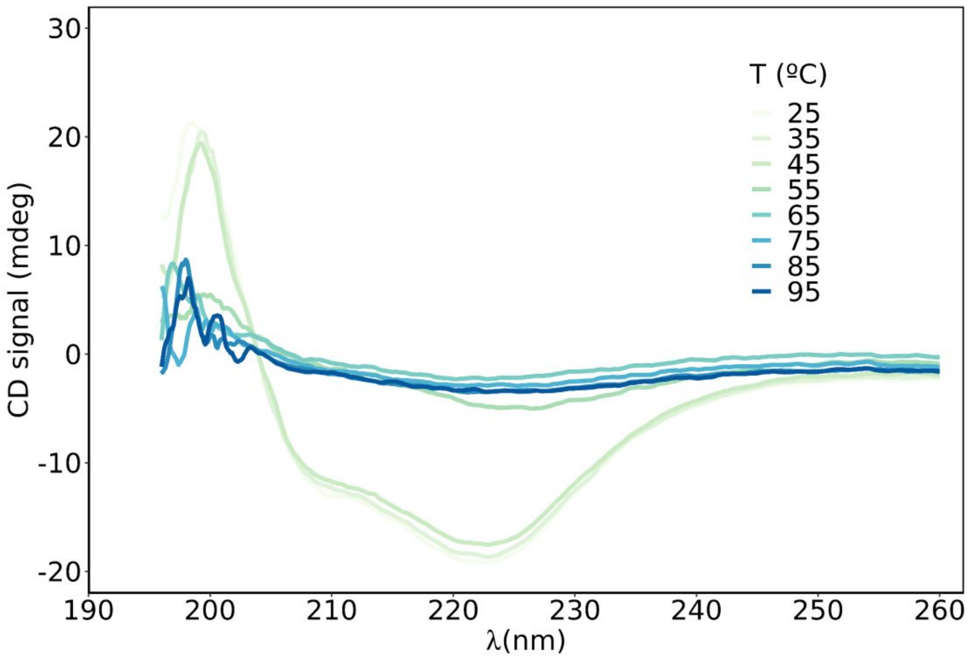

Figure S5. Biophysical Characterization of SARS-CoV-2 M^{pro}. Circular dichroism (CD) spectra of SARS-CoV-2 M^{pro} measured across wavelengths from 190 to 260 nm at temperatures ranging from 10°C, starting at 25°C and increasing incrementally to 95°C. The inset in the upper right corner provides a detailed description of the spectral changes corresponding to each temperature variation.

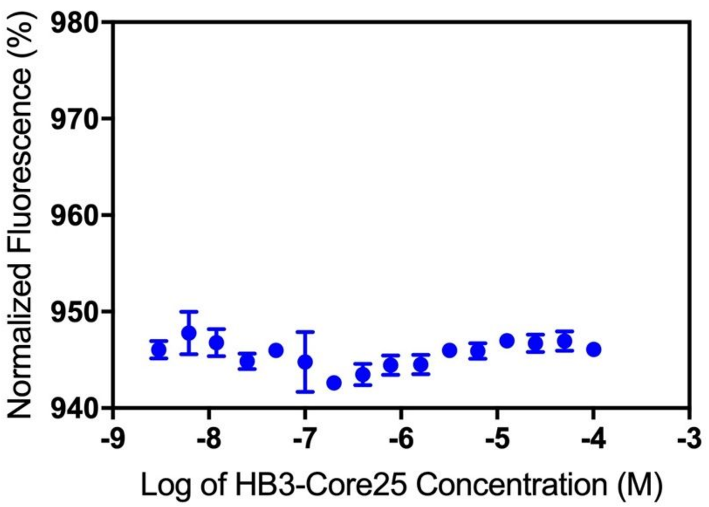

Figure S6. MST binding assay of double mutant E290A / R298A monomeric M^{pro}. Each point represents the change in fluorescence of the labeled homodimeric M^{pro} as the concentration of the HB3-Core25 inhibitor increases. No binding was observed, and therefore no dissociation constant has been determined.

Table S1. Metrics of the monomers and the interfaces between the mini-proteins and M^{pro}, as well as the known dimerization inhibitors of the M^{pro} from SARS-CoV-2: N8 peptide and VHH NB2B4. The following metrics are shown for the monomers: 1) Energy per residue; 2) Buried non-polar area (nspa) per residue; 3) Intraproteic shape complementarity (SS-Sc); Number of alanine residues in the hydrophobic core. For the interfaces, the following metrics are presented: 1) ddG = change in Rosetta energy of the binding partners when separated versus when complexed; 2) dG − separated /ΔS ASA ∗ 100 = Binding energy density; 3) ΔS ASA -interface = solvent-accessible surface area buried in the interface; 4) ΔS ASA -hydrophobic = solvent-accessible surface area buried in the interface that is hydrophobic; 5) ΔS ASA -polar = solvent-accessible surface area buried in the interface that is polar; 6) % of polar area in the interface: ΔS ASA −polar /ΔS ASA −interface; 7) Interface shape complementarity (Sc); 8) Packstat = Rosetta score for interface packing, where 0 (low packing) to 1 (high packing); 9) Molecular contact area (CMS); 10) Number of hydrogen bonds (HB) in the interface; 11) ΔS ASA Insat HB = number of buried and unsatisfied hydrogen bonds in the interface.

	Metrics	HB3-Core25	Mpro-N8	Mpro-VHH NB2B4
**Monomer**	Energy (reu/res)	-3.48		
	SS-Sc	60.72		
	NSPA	0.82		
	ddG (REU)	-61.14	-30.75	-60.87
**Interface**	dG-separeted/Δ SASA ∗ 100	-3.16	-3.64	-3.45
	ΔSASA-interface	1943	854	1760
	ΔSASA -hydrophobic	1014	438	965
	ΔSASA -polar	929	415	794
	% polar area at the interface	47.82	48.59	54.83
	Sc	0.71	0.82	0.66
	PackStat	0.7	0.72	0.7
	CMS	546	252	504
	Número de LH	18	10	15
	Δ Insat LH	8	2	9

Table S2. Percentages of interactions between amino acid residues in protein-protein complexes during molecular dynamics simulations. Only interactions with percentages equal to or less than 70% are included.

**Mpro homodimer**			**Mpro - HB3 core25**		
**Monomer A**	**Monomer B**	**%**	**HB3 core25**	**Mpro**	**%**
ALA7.A	VAL431.B	100	ARG30.A	GLU290.B	100
VAL125.A	ALA313.B	100	MET26.A	TYR126.B	100
ARG4.A	TYR432.B	99	ARG30.A	LYS85B	100
SER1.A	GLU472.B	99	GLN27.A	LYS5.B	98
TYR126.A	MET312.B	98	GLU20.A	MET6.B	97
MET6.A	TYR432.B	98	LEU36.A	LEU286.B	96
SER1.A	PHE446.B	95	GLU34.A	LYS137.B	93
VAL303.A	SER429.B	94	MET24.A	MET6.B	91
TYR126.A	ARG310.B	92	GLN39.A	LEU286.B	91
PHE305.A	PRO428.B	92	GLU46.A	ARG4.B	90
ARG4.A	MET432.B	90	GLN39.A	SER284.B	88
PHE305.A	ASP427.B	87	VAL23.A	MET6.B	86
TYR126.A	TYR313.B	86	MET24.A	ARG4.B	85
MET6.A	MET432.B	85	GLN39.A	ALA285.B	82
ARG4.A	PHE446.B	84	GLN27.A	GLN127.B	80
TYR126.A	ASP430.B	80	TYR31.A	GLU288.B	78
VAL5.A	GLU472.B	77	VAL23.A	ALA7.B	76
PHE305.A	ASP426.B	74	VAL23.A	VAL125.B	75

Rosetta Scripts

1. Backbone generation:

```
<ROSETTASCRIPTS>

<SCOREFXNS>
            <ScoreFunction name="SFXN1" weights="fldsgn_cen">
       <Reweight scoretype="cen_pair_motifs" weight="1"/>
       <Reweight scoretype="hbond_sr_bb" weight="1.0" />
       <Reweight scoretype="hbond_lr_bb" weight="1.0" />
       <Reweight scoretype="target_clash" weight="1000.0" />
       <Set target_clash_pdb="target.pdb" />
            </ScoreFunction>
</SCOREFXNS>

<RESIDUE_SELECTORS>
   <Layer name="core" select_core="true" select_boundary="false" select_surface="false" core_cutoff="4.8"
surface_cutoff="2.0" use_sidechain_neighbors="true"/>
</RESIDUE_SELECTORS>

<TASKOPERATIONS>
</TASKOPERATIONS>

<FILTERS>
            #Filtragem para o clash entre as hélice e o target
   <ScoreType name="clash_check" scorefxn="SFXN1" score_type="target_clash" threshold="2500"
confidence="1" />
   #Fitro p/avaliação do número de resíduos no core da proteína.
   <ResidueCount name="core_count" max_residue_count="1000" min_residue_count="0"
count_as_percentage="false" residue_selector="core" confidence="0" />
   <ResidueCount name="res_count" max_residue_count="9999" confidence="0"/>
   <CalculatorFilter name="core_percentage" equation="-1.0*core_count / res_count" threshold="-0.20"
confidence="1">
       <Var name="core_count" filter="core_count"/>
       <Var name="res_count" filter="res_count"/>
   </CalculatorFilter>
   # Filtros para características das hélices
   <HelixKink     name="hk1" blueprint="%%blueprint%%" confidence="-1" />
            <HelixPairing  name="hp1d" helix_pairings="1-3.P" dist="10.0" cross="15.0" align="5.0"
blueprint="%%blueprint%%" confidence="-1" output_type="dist"/>
            <HelixPairing  name="hp1c" helix_pairings="1-3.P" dist="10.0" cross="15.0" align="5.0"
blueprint="%%blueprint%%" confidence="-1" output_type="cross"/>

</FILTERS>

<TASKOPERATIONS>
</TASKOPERATIONS>

<MOVERS>
            <SetSecStructEnergies name="set_ssene1" scorefxn="SFXN1" blueprint="%%blueprint%%"
hh_pair="1-2.A"/>
   <BluePrintBDR name="bdr1" use_abego_bias="1" scorefxn="SFXN1" constraints_NtoC="-1.0"
blueprint="%%blueprint%%" />
            </ParsedProtocol>
</MOVERS>
```

```
<PROTOCOLS>
            <Add mover_name="set_ssene1" />
            <Add mover_name="bdr1" />
   <Add filter_name="clash_check" />
   <Add filter_name="core_percentage" />
   <Add filter_name="hp1c" />
   <Add filter_name="hp1d" />
   <Add filter_name="hk1" />
</PROTOCOLS>

</ROSETTASCRIPTS>
```

Blueprint file:

```
0 V LG R
0 V HA R
0 V HA R
0 V HA R
0 V HA R
0 V HA R
0 V HA R
0 V HA R
0 V HA R
0 V HA R
0 V HA R
0 V HA R
0 V HA R
0 V HA R
0 V HA R
0 V HA R
0 V LG R
0 V LB R
1 V HA R
2 V HA R
3 V HA .
4 V HA .
5 V HA .
6 V HA .
7 V HA .
8 M HA .
9 V HA .
10 V HA .
11 V HA .
12 R HA .
13 V HA .
14 V HA .
15 V HA .
16 V HA R
0 V LG R
0 V LB R
0 V HA R
0 V HA R
0 V HA R
0 V HA R
0 V HA R
```

```
0   V   HA   R
0   V   HA   R
0   V   HA   R
0   V   HA   R
0   V   HA   R
0   V   HA   R
0   V   HA   R
0   V   HA   R
0   V   HA   R
0   V   HA   R
0   V   LO   R
```

Flags

```
## Input:
-in:file:s motif.pdb

###Protocol
-parser:protocol denovo_hhh.xml
-parser:script_vars blueprint=H1H16H3LGB.bp
-nstruct 15000
-out:prefix H1H16H3LGB_

# O número de estruturas foi definido por tamanho da sequencia*300 (Baseado no artigo X)

## Best practice flags:
-ex1
-ex2
-use_input_sc
-no_his_his_pairE
-nblist_autoupdate true
-chemical:exclude_patches LowerDNA UpperDNA Cterm_amidation SpecialRotamer VirtualBB ShoveBB
VirtualDNAPhosphate VirtualNTerm CTermConnect sc_orbitals pro_hydroxylated_case1
pro_hydroxylated_case2 ser_phosphorylated thr_phosphorylated tyr_phosphorylated tyr_sulfated
lys_dimethylated lys_monomethylated  lys_trimethylated lys_acetylated glu_carboxylated cys_acetylated
tyr_diiodinated N_acetylated C_methylamidated MethylatedProteinCterm

## For ramping down constraints during relaxes:
-relax::ramp_constraints true

## Flags to reduce the verbosity of the output:
-mute core.pack.interaction_graph.interaction_graph_factory
-mute core.scoring.rms_util
-mute core.pack.task
-mute core.scoring.NeighborList
-mute core.pack.annealer.MultiCoolAnnealer
-mute core.pack.pack_rotamers
-mute protocols.forge.remodel.RemodelDesignMover

-staged_sampling
-use_same_length_fragments true
-picking_old_max_score 0.9
```

2. Monomer and interface sequence design:

```xml
<ROSETTASCRIPTS>
<SCOREFXNS>
<ScoreFunction name="ref2015" weights="ref2015"/>
</SCOREFXNS>
<RESIDUE_SELECTORS>
<!-- CHAINS -->
<Chain name="chainA" chains="A"/>
<Chain name="chainB" chains="B"/>
<ResidueName name="pro_and_gly_positions" residue_name3="PRO,GLY"/>
<!-- Layer Design -->
<Layer name="surface" select_core="false" select_boundary="false" select_surface="true"
use_sidechain_neighbors="true"/>
<Layer name="boundary" select_core="false" select_boundary="true" select_surface="false"
use_sidechain_neighbors="true"/>
<Layer name="core" select_core="true" select_boundary="false" select_surface="false"
use_sidechain_neighbors="true"/>
<SecondaryStructure name="sheet" overlap="0" minH="3" minE="2" include_terminal_loops="false"
use_dssp="true" ss="E"/>
<SecondaryStructure name="entire_loop" overlap="0" minH="3" minE="2" include_terminal_loops="true"
use_dssp="true" ss="L"/>
<SecondaryStructure name="entire_helix" overlap="0" minH="3" minE="2" include_terminal_loops="false"
use_dssp="true" ss="H"/>
<And name="helix_cap" selectors="entire_loop">
<PrimarySequenceNeighborhood lower="1" upper="0" selector="entire_helix"/>
</And>
<And name="helix_start" selectors="entire_helix">
<PrimarySequenceNeighborhood lower="0" upper="1" selector="helix_cap"/>
</And>
<And name="helix" selectors="entire_helix">
<Not selector="helix_start"/>
</And>
<And name="loop" selectors="entire_loop">
<Not selector="helix_cap"/>
</And>
</RESIDUE_SELECTORS>
<TASKOPERATIONS>
<!-- Layer Design -->
<DesignRestrictions name="layer_design">
<Action selector_logic="surface AND helix_start" aas="DEHKPQR"/>
<Action selector_logic="surface AND helix" aas="EHKQR"/>
<Action selector_logic="surface AND sheet" aas="EHKNQRST"/>
<Action selector_logic="surface AND loop" aas="DEGHKNPQRST"/>
<Action selector_logic="boundary AND helix_start" aas="ADEHIKLMNPQRSTVWY"/>
<Action selector_logic="boundary AND helix" aas="ADEHIKLMNQRSTVWY"/>
<Action selector_logic="boundary AND sheet" aas="DEFHIKLMNQRSTVWY"/>
<Action selector_logic="boundary AND loop" aas="ADEFGHIKLMNPQRSTVWY"/>
<Action selector_logic="core AND helix_start" aas="AFILMPVWY"/>
<Action selector_logic="core AND helix" aas="AFILMVWY"/>
<Action selector_logic="core AND sheet" aas="FILMVWY"/>
<Action selector_logic="core AND loop" aas="AFGILMPVWY"/>
<Action selector_logic="helix_cap" aas="DNST"/>
</DesignRestrictions>
<!-- Interface -->
<ProteinInterfaceDesign name="pido" repack_chain1="1" repack_chain2="1" design_chain1="1"
design_chain2="0" interface_distance_cutoff="8.0"/>
<RestrictToInterfaceVector name="rtiv" chain1_num="1" chain2_num="2" CB_dist_cutoff="10.0"
nearby_atom_cutoff="5.5" vector_angle_cutoff="75" vector_dist_cutoff="9.0"/>
<!-- Repack -->
<RestrictToRepacking name="rtr"/>
```

```
<PreventResiduesFromRepacking name="prfrp" residues="%%hotspot%%"/>
<InitializeFromCommandline name="init"/>
<!-- Restrict residues  -->
<DisallowIfNonnative name="disallow_GLY" resnum="0" disallow_aas="G"/>
<DisallowIfNonnative name="disallow_PRO" resnum="0" disallow_aas="P"/>
<OperateOnResidueSubset name="restrict_PRO_GLY" selector="pro_and_gly_positions">
<PreventRepackingRLT/>
</OperateOnResidueSubset>
<!-- Restrict ChainB  -->
<OperateOnResidueSubset name="restrict_target" selector="chainB">
<PreventRepackingRLT/>
</OperateOnResidueSubset>
<PreventResiduesFromRepacking name="restrict_target2" residues="%%target_contact%%"/>
</TASKOPERATIONS>
<FILTERS>
<!-- Interface Metrics -->
<Sasa name="interface_buried_sasa" confidence="0"/>
<ShapeComplementarity name="Sc" min_sc="0" write_int_area="1" jump="1" confidence="0"/>
<Ddg name="ddg" scorefxn="ref2015" threshold="0" jump="1" repeats="5" repack="1" repack_bound="0"
confidence="0"/>
<ContactMolecularSurface name="contact_area_target" verbose="0" distance_weight="0.5" confidence="0"
target_selector="chainB" binder_selector="chainA"/>
</FILTERS>
<MOVERS>
<!-- FastDesign monomer -->
<FastDesign name="FDesign_monomer" scorefxn="ref2015" repeats="5"
task_operations="init,layer_design,prfrp,disallow_GLY,disallow_PRO,restrict_PRO_GLY,restrict_target,restr
ict_target2" batch="false" ramp_down_constraints="false" cartesian="false" bondangle="false"
bondlength="false" min_type="dfpmin_armijo_nonmonotone"/>
<!-- FastDesign interface -->
<FastDesign name="FDesign_interface" scorefxn="ref2015" repeats="5"
task_operations="init,layer_design,prfrp,disallow_GLY,disallow_PRO,restrict_PRO_GLY,pido,rtiv,restrict_t
arget,restrict_target2" batch="false" ramp_down_constraints="false" cartesian="false" bondangle="false"
bondlength="false" min_type="dfpmin_armijo_nonmonotone"/>
<!-- FastRelax -->
<FastRelax name="FastRelax" scorefxn="ref2015" repeats="1" batch="false" ramp_down_constraints="false"
cartesian="false" bondangle="false" bondlength="false" min_type="dfpmin_armijo_nonmonotone"
task_operations="init,rtr,rtiv,prfrp,restrict_target2"/>
<!-- Interface Analyzer -->
<InterfaceAnalyzerMover name="ifa" scorefxn="ref2015" packstat="1" pack_separated="1" pack_input="1"
tracer="0" interface_sc="1" interface="A_B"/>
</MOVERS>
<PROTOCOLS>
<!--Design Protocol-->
<Add mover="FDesign_monomer"/>
<Add mover="FDesign_interface"/>
<Add mover="FastRelax"/>
<!--Interface Analyzer-->
<Add mover="ifa"/>
<!--Filters interface-->
<Add filter="interface_buried_sasa"/>
<Add filter="Sc"/>
<Add filter="contact_area_target"/>
<Add filter="ddg"/>
</PROTOCOLS>
<OUTPUT scorefxn="ref2015"/>
</ROSETTASCRIPTS>
```

Rosetta flags:

```
-in:file:s HB3.pdb
-use_input_sc
-parser:protocol fastdesign.xml
-parser:script_vars hotspot=26A,30A   #"46B,49B,53B,55B,91B,94B,96B"
#-parser:script_vars target_contact=
-ex1                                                  # increase rotamer bins to include mean +- 1
standard deviation
-ex2                                                  # increase rotamer bins to include mean +- 2
standard deviations
-ex2aro
-nstruct 60000
-out:file:scorefile HB3_fastdesign.sc
```
